# Recent advances of lysine lactylation in prokaryotes and eukaryotes

**DOI:** 10.3389/fmolb.2024.1510975

**Published:** 2025-01-09

**Authors:** Wenjuan Zhao, Jiayi Xin, Xin Yu, Zhifang Li, Nan Li

**Affiliations:** ^1^ School of Pharmacy, Faculty of Medicine, Macau University of Science and Technology, Macau, China; ^2^ Shenzhen Key Laboratory of Genome Manipulation and Biosynthesis, Key Laboratory of Quantitative Synthetic Biology, Shenzhen Institute of Synthetic Biology, Shenzhen Institutes of Advanced Technology, Chinese Academy of Sciences, Shenzhen, China; ^3^ School of life sciences, Henan University, Kaifeng, China

**Keywords:** lysine lactylation, post-translational modification, lactate, cell metabolism, tumorigenesis

## Abstract

Lysine lactylation is a newly discovered protein post-translational modification that plays regulatory roles in cell metabolism, growth, reprogramming, and tumor progression. It utilizes lactate as the modification precursor, which is an end product of glycolysis while functioning as a signaling molecule in cells. Unlike previous reviews focused primarily on eukaryotes, this review aims to provide a comprehensive summary of recent knowledge about lysine lactylation in prokaryotes and eukaryotes. The current identification and enrichment strategies for lysine lactylation are introduced, and the known readers, writers, and erasers of this modification are summarized. In addition, the physiological and pathological implications of lysine lactylation are reviewed for different organisms, especially in prokaryotic cells. Finally, we end with a discussion of the limitations of the studies so far and propose future directions for lysine lactylation investigations.

## 1 Introduction

Lysine lactylation is an emerging post-translational modification (PTM) that influences a variety of cellular processes, including cell metabolism (Dong et al., 2022; Li et al., 2023), neuronal development (Dai et al., 2022; Merkuri et al., 2024), cellular reprogramming (Li L. et al., 2020; Hu et al., 2024), inflammation (Yang et al., 2022; Fan M. et al., 2023), and tumorigenesis (Xie et al., 2024; Zong et al., 2024). This PTM has three distinct isomers: L-lactylation (K_L-la_), D-lactylation (K_D-la_), and N-ε-(carboxyethyl)-Lysine (K_ce_) (Zhang et al., 2024). The cellular levels of K_L-la_ and K_D-la_ can be stimulated by the two optical isomers of lactate, i.e., L-lactate and D-lactate, respectively. As a byproduct of cellular metabolism (Cornell et al., 1974; Ewaschuk et al., 2005; Vander Heiden et al., 2009), lactate serves as a critical signaling molecule, regulating tumor development and immune responses (Certo et al., 2021; Zhang et al., 2022; Li H. et al., 2024). Given its broad biological relevance, it is vital to understand the mechanisms behind lysine lactylation, for exploring its physiological and pathological roles.

Recently studies suggest that lysine lactylation has mechanistic similarities with lysine acylation, which has been studied extensively since the 1960s (Allfrey et al., 1964; Ren et al., 2017; Gong et al., 2024). To introduce and remove the modification, there are generally two distinct types of catalytic mechanisms, enzymatic and non-enzymatic. The enzymatic mechanism is conducted by lactyltransferases (writers) and delactylases (erasers), which function similarly to acetyltransferases (KATs) and deacetylases. K_L-la_ is tightly regulated by these enzymes, which can install and remove the L-lactyl groups rather than the acetyl group to lysine residues (Li et al., 2023; Sung et al., 2023; Zhang et al., 2024). These enzymes have been extensively studied in eukaryotes (Chen et al., 2024; Li H. et al., 2024), while much less studies have been conducted in prokaryotes (Dong et al., 2022; Li et al., 2023; Zong et al., 2024). Studies in *Escherichia coli* (*E. coli*) and *Streptococcus mutans* (*S. mutans*) have shown that, lysine lactylation is a PTM conserved across prokaryotes and eukaryotes, much like lysine acetylation (Hentchel and Escalante-Semerena, 2015; Wei et al., 2017). Furthermore, several lactyltransferases and delactylases have been identified in various microbial species. On the other hand, a novel nonenzymatic mechanism has been identified in HEK293 cells (Gaffney et al., 2020). The authors reported that, S-D-lactylglutathione (LGSH) directly donates its D-lactyl group to lysine residues, generating K_D-la_ modification. However, far less has been known for this form of lactylation.

These emerging findings suggest that, exploring the functional significance of lysine lactylation, in physiological and pathological cellular contexts, offers a promising direction in chemical and cell biology. Understanding these mechanisms could provide new insights into disease regulation and therapeutic development. In this review, we first introduce the lactylation discovery history, followed by a summary of detection methods of lysine lactylation. We then comprehensively explore the regulatory enzymes of lactylation (writers, erasers, and readers), along with the physiological and pathological roles of lactylation across various organisms. Finally, we discuss the current challenges and limitations in lactylation research and propose potential directions for future studies. Ultimately, this review aims to deepen our understanding of lysine lactylation and uncover new therapeutic strategies for lactylation-related diseases.

## 2 Discovery of lysine lactylation

### 2.1 Discovery history

**FIGURE 1:**
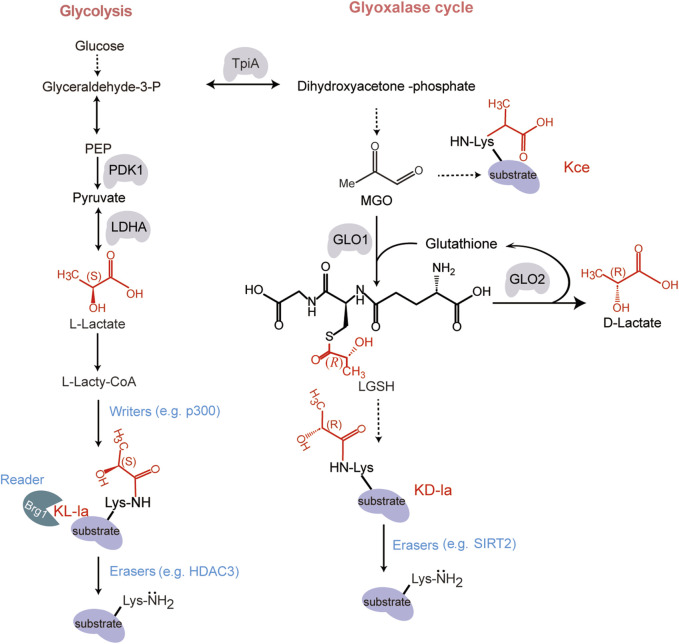
The structure and formation mechanism of K_L-la_, K_D-la_ and K_ce._ The formation of these chemical structural isomers of K_L-la_, K_D-la_ and K_ce_ are produced through two pathways: glycolysis and the glyoxalase cycle. During glycolysis, a series of enzymatic reactions transpire within the cytoplasmic compartment of the cell. Glucose undergoes a step-by-step degradation through a series of intermediate reactions, culminating in the production of lactate (Vander Heiden et al., 2009). The lactate exists in two stereoisomeric forms: L-lactate [with (*S*) configuration] and D-lactate [with (*R*) configuration]. K_L-la_ is induced by glycolysis-derived L-lactate, which involves the transfer of an L-lactyl group from an L-lactyl-CoA to a lysine residue on a target protein, which is regulated by the writer p300 and eraser HDAC3 (Zhang et al., 2019; Zhang et al., 2024). K_D-la_ is formed by an uncatalyzed reaction involving LGSH generation through the glyoxalase cycle pathway, as detailed by Gaffney and colleagues (Gaffney et al., 2020). K_ce_, another lysine lactylation isomer, is one of the MGO adducts (Khadha et al., 2020; Kulkarni and Brookes, 2020). Designed and created by Wenjuan Zhao and Jiayi Xin.

In 2019, Zhang and colleagues discovered K_L-la_ that is driven by L-lactate (Zhang et al., 2019). Using high-performance liquid chromatography (HPLC)-tandem mass spectrometry (MS/MS) analysis, they observed a mass shift of 72.021 Da on lysine residues in histone proteolytic peptides. The mass shift corresponds precisely to the addition of a lactyl group to the ε-amino group of a lysine residue. Subsequent immunoblotting, metabolic labelling and MS/MS analysis demonstrated that lysine lactylation is derived from L-lactate (Zhang et al., 2019) (Figure 1). These findings demonstrate that lactylation specifically occurs on lysine residues of histones.

Following the discovery of K_L-la_, Gaffney et al. identified its stereoisomer, K_D-la_, which occurs on both histones and non-histone proteins (Gaffney et al., 2020). K_D-la_ is formed via a nonenzymatic reaction by transferring a D-lactyl group from LGSH to lysine residue (Gaffney et al., 2020; Trujillo et al., 2024). LGSH is produced through the glyoxalase pathway, which involves two enzymes: glyoxalase 1 (GLO1) and glyoxalase 2 (GLO2). GLO1 catalyzes the reaction between the glycolysis byproduct methylglyoxal (MGO) and glutathione (GSH) to form LGSH. GLO2 hydrolyzes LGSH to produce D-lactate and regenerate GSH (Figure 1). Subsequent studies showed that K_D-la_ increased in histone H4 when incubated with LGSH. This result was reproducible with the glycolytic enzyme phosphoglycerate kinase 1 (PGK1). These research findings indicate that LGSH levels are the primary driver of K_D-la_ formation in cells. Additionally, MGO can directly react with lysine, generating *N*-ε-(carboxyethyl)-lysine (K_ce_), another isomer of lysine lactylation (Galligan et al., 2018; Khadha et al., 2020; Kulkarni and Brookes, 2020; Zhang et al., 2024) (Figure 1).

### 2.2 Identification and enrichment strategies

**FIGURE 2:**
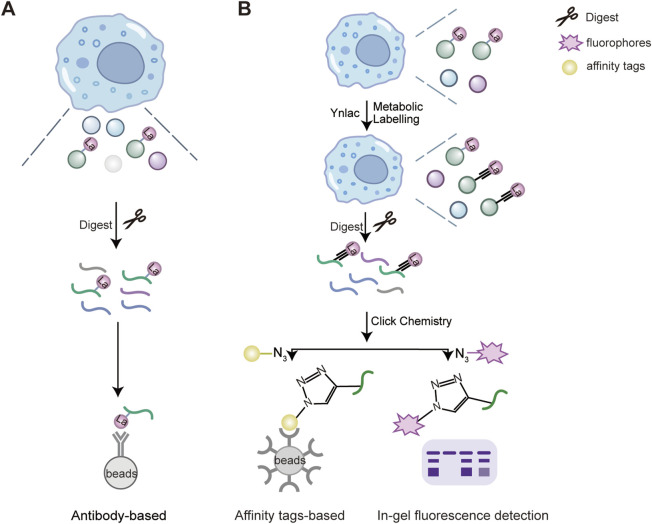
Proteomics strategies for the detection of lysine lactylation. The enrichment and detection methods of lactylated proteins include a pan-anti-lactylation antibody-based strategy **(A)** and a biorthogonal chemical reporter **(B)**. **(A)** The pan-anti-lactylation antibody are derivatized to be immobilized on the solid support and incubated with cell lysates. Lysine lactylation modified peptides after tryptic digestion are enriched and pulled down on solid supports. **(B)** Bioactive compounds are functionalized with photoaffinity linkers such as the alkynyl derivatives of PTM donor precursors, such as YnLac, alkynyl-functionalized L-lactate analogue. All newly synthesized lactylated proteins can be metabolically labelled with YnLac in cell culture. Then the labelled proteins are digested by trypsin and conjugated with fluorescent or affinity tags with azide-coated through Click chemistry. Next, the labelled peptide are enriched for in-gel fluorescence detection or pulled down through affinity tags immobilized solid support (Sun et al., 2022a). Designed and created by Wenjuan Zhao and Jiayi Xin.

A variety of chemical tools have been developed to investigate lysine lactylation at the proteomics level. These include specific antibodies for enriching lactylation peptides and bio-orthogonal chemical probes for metabolic labeling (Figure 2). In 2019, Zhang and colleagues introduced a pan anti- K_L-la_ antibody-based immunoprecipitation method, combined with MS/MS to identify target proteins and modification sites of K_L-la_ in cells (Figure 2A) (Zhang et al., 2019). This technique has been used in numerous studies to examine the presence of K_L-la_ in various eukaryotic organisms, including Kupffer cells (Sung et al., 2023), *Botrytis cinerea* (Gao et al., 2020), maize root (Shi et al., 2023), and *Caenorhabditis elegans* (Ding et al., 2024). In 2024, they developed second-generation antibodies that effectively distinguish between K_L-la_, K_D-la_, and K_ce_.

Bio-orthogonal chemical analogs has emerged as powerful tools for metabolic labeling and proteomic analysis of PTMs (Stone et al., 2017; Gao and Hannoush, 2018; Parker and Pratt, 2020). These analogs, such as the alkynyl or azido derivatives of PTM donor precursors, are metabolically incorporated into lactylated proteins. Then labelled proteins are conjugated with fluorophores or affinity tags through click chemistry for subsequent fluorescence detection or proteomic analysis (Parker and Pratt, 2020). In 2022, Sun and colleagues developed YnLac, an alkynyl-functionalized bio-orthogonal analog of L-lactate, for profiling of protein lactylation (Figure 2B) (Sun et al., 2022a). This study not only identified lactylation sites on histones but also revealed novel lactylation sites on non-histone proteins, such as nucleolar protein NCL, DNA chaperone HMGB1 and poly (ADP-ribose) polymerase 1 (PARP1). For example, lactylation of PARP1 may regulate its ADP-ribosylation activity, which is essential for its role in DNA repair, indicating a potential regulatory role for lactylation in DNA repair mechanisms (Sun et al., 2022a).

In addition to experimental approaches, computational prediction tools have become a cost-effective ways to identify potential modification sites. In 2021, Jiang et al. introduced the first predictive model for lysine lactylation sites, called FSL-Kla (Jiang et al., 2021). After this, more models have been developed, such as DeepKla and Auto-Kla (Lv et al., 2022; Lai and Gao, 2023). More recently, Yang et al. developed two enhanced frameworks, ABFF-Kla and EBFF-Kla, which integrate both protein sequences and 3D structural features, providing the accuracy of prediction (Yang et al., 2024). These tools improve our ability to identify lactylation sites and offer deeper insights into the substrate proteins and the biological processes they regulate.

## 3 Writer, eraser and reader of lactylation

### 3.1 Writer

Recent studies have identified KATs as key writers that regulate K_L-la_. KATs can be classified into three families: the p300/CBP, the MYST, and the GNAT (Berndsen and Denu, 2008; Friedmann and Marmorstein, 2013). The p300/CBP and MYST families are involved in regulating K_L-la_ in eukaryotes (Table 1). For example, the absence or inhibition of p300/CBP was shown to reduce the lactylation level of proteins such as HMGB1 and MRE11 (Yang et al., 2022; Chen et al., 2024). Yu et al. demonstrated that P300 depletion could reduce the lactylation level of histone, disrupting the binding of P300 to YTHDF2 promoter, and affecting gene expression (Yu et al., 2021). Members of MYST family, KAT8/MOF and KAT5/TIP60, were shown to regulate K_L-la_ in non-histone proteins such as Vps34 and eEF1A2 (Jia et al., 2023; Xie et al., 2024). These findings indicate that p300/CBP and MYST families serve as writers for both histone and non-histone lactylation.

**TABLE 1:** Summary of the writers, readers, and erasers of lysine lactylation and their corresponding sites and functions under different conditions.

Type	Enzyme	Lactylated		Regulated proteins	Result	Ref.
		Proteins	Sites			
Writers	P300	H3	K18	ARG 1	Induces M2- like genes transcription in M1 macrophages	Zhang et al. (2019)
				YTHDF 2	Promotes YTHDF2’s transcription to accelerate tumorigenesis	Yu et al. (2021)
				METTL 3	Enhances the capture of m6A-modified RNA to promote immunosuppression	Xiong et al. (2022)
				Oct 4 Sall4 Mycn	Facilitates cellular reprogramming	Li et al. (2020a)
				Cdh 1 Oct 4	Activate pluripotent genes and promotes iPSC reprogramming	Hu et al. (2024)
				circATXN7	Activates transcription of circATXN7 to foster tumor immunoescape	Zhou et al. (2024)
		H4	K12	PKM2	Forms a positive feedback glycolysis/H4K12la/PKM2 loop, exacerbates microglial dysfunction	Pan et al. (2022)
		YY1	K183	FGF2	Upregulates FGF2 and promotes angiogenesis	Wang et al. (2023)
		AK2	K28	-	Weakens AK2 enzymatic activity and contributes to HCC malignancy	Yang et al. (2023b)
	P300/CBP	Snail1	-	TGF-β	Promotes endothelial-to-mesenchymal transition after myocardial infarction	Fan et al. (2023a)
		HMGB1	-	-	Promotes Hmgb1 translocation from the nucleus to the cytoplasm and its release	Yang et al. (2022), Du et al. (2023)
	CBP	MRE11	K673	-	Promotes DNA end resection and HR repair	Chen et al. (2024)
	GCN5	H3	K18	*Lrg1* *Vegf-a* *IL-10*	Boosts reparative gene activation post–myocardial infarction	Wang et al. (2022)
	KAT5 (TIP60)	Vps34	K356 K781	Vps34	Activates Vps34 lipid kinase activity in muscle cells and cancer cells	Jia et al. (2023)
	KAT8	eEF1A2	K408	-	Promotes eEF1A2-mediated protein synthesis and colorectal carcinogenesis	Xie et al. (2024)
	AARS2	PDHA1	K336	-	Limits oxidative phosphorylation	Mao et al. (2024)
		CPT2	K457 K458			
	AARS1/	p53	K120K139	-	Impairs p53 LLPS and DNA binding, thereby reducing p53 tumor-suppressive roles	Zong et al. (2024)
	YiaC	GltA PncB	K328K381	-	Regulates the activity of metabolic enzymes	Dong et al. (2022)
	GNAT13	RpoA	K173	-	Inhibits the synthesis of extracellular polysaccharides	Li et al. (2023)
Erasers	SIRT1	non-histone proteins		-	A potential delactylase for non-histone proteins	Sun et al. (2022b)
	SIRT2	LactoylLys			Regulates Protein Lactoyl-Lys Modifications	Jennings et al. (2021)
		H4	K8	SERPING1 TRPV4	Inhibits the proliferation and migration of neuroblastoma cells	Zu et al. (2022)
	SIRT3	H4	K16	-	-	Fan et al. (2023b)
		cyclin E2	K348	-	Delactylation of cyclin E2 prevents hepatocellular carcinoma growth	Jin et al. (2023)
		Fis1	K20	-	Reducing Fis1 lactylation attenuates sepsis-induced acute kidney injury	An et al. (2023)
		PDHA1	K336	-	Decreases PDHA1 and CPT2 lactylation to promote OXPHOS	Mao et al. (2024)
		CPT2	K457 K458	-		
	HDAC1-3	H3 H4	K18 K5	-	Influences H3K18la levels and regulates neural differentiation of P19 EC cells	Dai et al. (2022), Moreno-Yruela et al. (2022), Zessin et al. (2022)
	CobB	PykF	K382	-	Regulates PykF activity and influences cell growth	Dong et al. (2022)
Readers	Brg1	H3	K18	-	Brg1 works as a reader of H3K18la in early reprogramming	Hu et al. (2024)

The GNAT family of acetyltransferases is present in both eukaryotes and prokaryotes (Table 1) (Hentchel and Escalante-Semerena, 2015; Burckhardt and Escalante-Semerena, 2020). Recent studies found that the members of this family, like GCN5, YiaC, and GNAT13, play a role in lysine lactylation (Dong et al., 2022; Wang et al., 2022; Li et al., 2023). Wang and colleagues showed that silencing GCN5 in cells lead to a significant reduction in histone K_L-la_ (Wang et al., 2022). In prokaryotes, Dong et al. identified 79 potential K_L-la_ sites regulated by YiaC (Dong et al., 2022). Li et al. also demonstrated that GNAT13 catalyzes lysine lactylation in *S. mutans* (Li et al., 2023).

In addition to KATs, the aminoacyl-tRNA synthetase (AARS) family has recently been found to catalyze lysine lactylation (Table 1). Mao et al. showed that mitochondrial AARS2 is a lysine lactyltransferase, adding lactyl groups to PDHA1 and CPT2 (Mao et al., 2024). Similarly, Zong et al. demonstrated that cytoplasmic AARS1 in tumor cells binds lactate and catalyzes lactylation of p53 at K120 and K139 residues. Recent studies have identified that AARS1/2 are conserved intracellular sensors of L-lactate and play an essential role as lactyltransferases to stimulate the lysine lactylome in cells (Li H. et al., 2024; Zong et al., 2024).

### 3.2 Eraser

Lysine deacetylases (KDACs) are enzymes that remove acyl groups from lysine residues. They include histone deacetylases (HDACs), which are Zn^2+^ dependent, and sirtuins (SIRTs), which require NAD^+^ as a co-substrate (Table 1) (Tan et al., 2016; Moreno-Yruela et al., 2022; Yang Y. et al., 2023). Moreno-Yruela et al. screened all 18 HDACs to evaluate their ability to cleave ε-N-L-lactyllysine and found that HDAC1-3 and SIRT1-3 exhibit delactylase activity *in vitro*. Among them, HDAC1-3 are the most efficient enzymes for removing lactyl groups from lysine residues (Moreno-Yruela et al., 2022). Meanwhile, Zu and colleagues discovered SIRT2 as a key eraser of histone lysine lactylation, which inhibits the proliferation and migration of glioblastoma cells (Zu et al., 2022). Furthermore, research by Zessin’s group revealed that HDAC3 is the most effective delactylase for histones. Its delactylase activity is over 1,000 times more than SIRT2 or other HDAC isoforms (Zessin et al., 2022).

In addition to these findings, Sun et al. utilized genetic code expansion technology to identify delactylases in both bacteria and mammalian cells. Their study identified SIRT1 as a potential delactylase for non-histone proteins (Sun et al., 2022b). Similarly, Jennings and colleagues demonstrated that SIRT2 removes K_D-la_ from protein pyruvate kinase M2 (PKM2) (Jennings et al., 2021). Recent studies have highlighted SIRT3 as the most effective eraser for non-histone lysine lactylation targets, such as Fis1 and CCNE2 (An et al., 2023; Jin et al., 2023; Mao et al., 2024). However, there is still some debate regarding which enzyme should be considered the primary non-histone delactylase.

In prokaryotes, the most studied lysine deacetylase is the sirtuin 2–like protein CobB, found in *Salmonella* and *E. coli* (Starai et al., 2002; Zhao et al., 2004; Yang Y. et al., 2023). CobB serves as the primary deacylase in *E. coli*, responsible for removing acetylation and succinylation modifications (Zhao et al., 2004). Dong et al. recently confirmed that CobB also functions as an endogenous lysine delactylase in *E. coli*. Their quantitative proteomics analysis identified 446 endogenous K_L-la_ sites in *E. coli*, regulated by CobB, highlighting its role in lysine lactylation dynamics in prokaryotes (Dong et al., 2022).

### 3.3 Reader

To date, the study of lysine lactylation readers is still in its early stages. So far only one study has identified a specific reader: bromodomain-containing protein Brg1 (Table 1). Hu et al. demonstrated that Brg1 interacts with H3K18la, influencing cell reprogramming by modulating chromatin structure and gene expression (Hu et al., 2024). While this discovery provides a foundation, the broader research of K_L-la_ readers and their regulatory roles remains largely unexplored.

These finding highlights the importance of understanding the proteins that regulate lysine lactylation, including its writers, erasers, and readers. However, our understanding of these mechanisms is still in its infancy. Continued investigation is needed to elucidate the regulatory network of these proteins and their biological significance in lysine lactylation.

## 4 Physiological and pathological regulation of lactylation

### 4.1 Cell growth and metabolic regulation in prokaryotes

**FIGURE 3:**
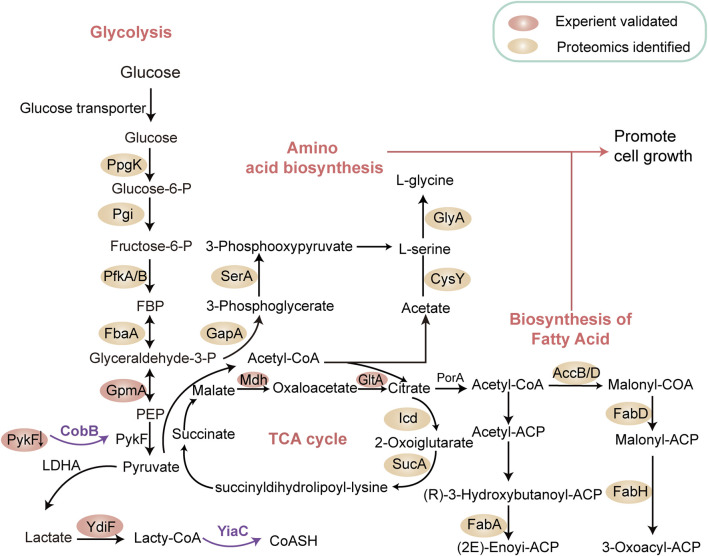
Lysine Lactylation modulates Metabolic Pathways in Prokaryotes. This schematic illustrates our current understanding of how metabolic pathways in prokaryotes are interconnected and directly regulated by lactylated enzymes within glycolysis, the tricarboxylic acid (TCA) cycle, nucleotide synthesis, and fatty acid biosynthesis networks. YiaC functions as a writer to catalyze the addition of K_L-la_, while CobB serves as an eraser to remove this lysine lactylation. The proteins in cycles represent the substrates for CobB or YiaC identified by proteomics. The pink cycles represent the substrates validated by experiment. Notably, YiaC was shown to enhance the lysine lactylation of GltA and PncB. CobB shows clear lysine lactylation eraser activity of PykF, GpmA, and Mdh. The removal of lactylation by CobB enhanced the enzymatic activity of PykF, thereby promoting glycolysis and bacterial growth (Dong et al., 2022). Designed and created by Wenjuan Zhao and Jiayi Xin.

Lactate, an important carbon source in prokaryotic metabolism (Jiang et al., 2014), influences various cellular processes (Brown et al., 2014). The discovery of lysine lactylation has broadened the biological significance of this metabolite. For instance, Dong et al. profiled the K_L-la_ proteome in *E. coli,* revealing widespread lysine lactylation in bacteria. The study identified YiaC functions as a writer enzyme that catalyzes the addition of K_L-la_ using lactyl-CoA, while CobB, an NAD + -dependent eraser, removes this PTM. Additionally, YdiF was found to catalyze lactyl-CoA formation in *E. coli* (Dong et al., 2022). Using quantitative proteomic analyses, they identified 1,047 K_L-la_ sites across 478 proteins candidates in *E. coli*. Among these, YiaC regulated 79 KL-la sites, while CobB targeted 446 sites. Gene Ontology analysis indicated that K_L-la_ modified proteins were enriched in pathways such as glycolysis, the TCA cycle, and fatty acid biosynthesis (Figure 3). Notably, YiaC- mediated lactylation on citrate synthase (GltA) and nicotinic acid phosphoribosyltransferase (PncB) suppressed enzymatic activity. In contrast, CobB-mediated delactylation of pyruvate kinase I (PykF) at K382 enhanced enzymatic activity, promoting glycolysis and growth (Figure 3) (Dong et al., 2022). Further studies by Zong et al. identified the lysine lactylome in *E. coli*, showing substantial overlap with lactylation patterns observed in mammalian cells, suggesting potential evolutionary conservation (Zong et al., 2024). This analysis revealed 4,544 K_L-la_ sites across 1,704 proteins in *E. coli.* Functional analysis indicated that K_L-la_ -modified proteins were predominantly involved DNA and RNA processes.

### 4.2 Bacterial virulence and pathogenicity regulation in prokaryotes

Lactate also serves as a substrate for numerous pathogenic microbes and has been shown to contribute to their pathogenicity. *S. mutans*, a facultative anaerobe, ferments host dietary carbohydrate to produce large amounts of L-lactate, a key virulence factor linked to its cariogenicity (Aires et al., 2008; Li et al., 2023). Li and colleagues proposed that *S. mutans*-derived lactate influences lysine lactylation and regulates various physiological functions associated with its cariogenic potential. Their study demonstrated that K_L-la_ at lysine 173 of the RNA polymerase subunit α (RpoA) decreased under high-sugar conditions. This reduction enhanced the synthesis of exogenous polysaccharides (EPSs), critical components of cariogenic biofilm, by inducing the expression of glucosyltransferases (Gtfs) and levansucrase (Ftf). Additionally, the study identified GNAT13 as responsible for increasing K_L-la_ at lysine 173 of RpoA. This dynamic adjustment enables S. mutans to rapidly form cariogenic biofilms in high-sucrose environments, accelerating enamel erosion (Li et al., 2023). Beyond dental caries, *S. mutans* has also been implicated in bacterial endocarditis, cerebral hemorrhage, and atherosclerosis. Investigating the role of K_L-la_ lactylation in *S. mutans* and its contribution to these pathologies hold significant potential for advancing prevention and treatment strategies.

Further research by Wang et al. revealed that lysine lactylation occurs in secreted proteins of the human pathogen *Staphylococcus aureus* (*S. aureus*). This modification, driven by lactate concentration, particularly affects alpha-toxin, a key virulence factor in *S. aureus* infections. Lactylation at lysine 84 of alpha-toxin was found to be essential for its full activity and virulence in infection models. Notably, extracellular lactate levels typically rise during infections, suggesting that pathogenic bacteria may use protein lactylation to enhance toxin-mediated virulence (Wang et al., 2024). These findings not only highlight a novel mechanism by which *S. aureus* adapts to the host environment, but also point out the potential of targeting lactylation enzymes in *S. aureus* and other pathogens as a strategy for anti-virulence therapy.

### 4.3 Neuronal development

**FIGURE 4:**
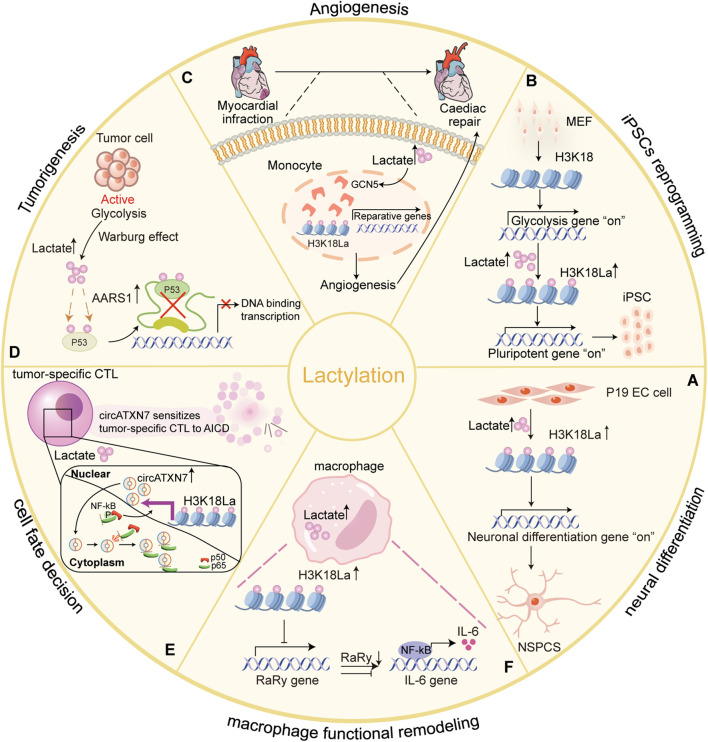
Lysine lactylation influences physiological and pathological processes by regulating gene expression and protein function in eukaryotes. Lactate acts as a signaling molecule to affect gene transcription and protein function via histone and non-histone lysine lactylation, and participates in physiological and pathological processes. **(A)** Histone H3K18 lactylation are tightly correlated with chromatin state and gene expression, and extensively involved in transcriptome remodeling associated with neuronal differentiation in differentiating embryonic carcinoma cells (P19 EC) cells and progenitor cells (NSPCs) (Dai et al., 2022); **(B)** Glis1 or Dux increases histone lactylation (H3K18la) at pluripotency loci, activating pluripotent genes and improving the efficiency of iPSC reprogramming (Li L. et al., 2020; Hu et al., 2024); **(C)** Increased H3K18 lactylation levels facilitated transcription of cardiac injury reparative genes in monocytes-macrophages early post-MI (Wang et al., 2022); **(D)** AARS1 as an intracellular L-lactate sensor and lactyltransferase that mediates global lysine lactylation and lactylation of p53 attenuates DNA binding and transcriptional activation, contributing to tumorigenesis (Zong et al., 2024); **(E)** In the tumor-specific CTLs, H3K18 lactylation induced by tumor cell produced lactic acid promotes circATXN7 expression, making tumor-specific CTLs sensitive to tumor-mediated AICD by binding to NF-κB p65 subunit and masking the p65 nuclear localization signal motif, thereby sequestering it in the cytoplasm (Zhou et al., 2024); **(F)** Histone lactylation inhibits RARg expression in macrophages, consequently enhancing IL-6 levels in the TME and endowing macrophages with tumor-promoting functions (Li X.-M. et al., 2024). Designed and created by Wenjuan Zhao and Jiayi Xin.

K_L-la_, the predominant form of lysine lactylation in humans and other eukaryotes (Zhang et al., 2024), plays a pivotal role in regulating neuronal development (Figure 4). In 2022, Dai et al. found that histone lactylation govern gene expression and facilitate transcriptome remodeling during neural development. Using an *in vitro* P19 cell neural differentiation system, they showed that inhibiting HDAC1-3 activity triggered a cascade of histone lysine acylation, pre-activating the neuron-specific transcription program (Figure 4A) (Dai et al., 2022). Similarly, Merkuri et al. explored the role of histone K_L-la_ in neural crest cells (NCC) differentiation. They observed that histone K_L-la_ was enriched on active enhancers of developmental genes within NCC gene regulatory networks (GRNs). This modification not only induced gene expression but also enhanced chromatin accessibility at these regions. Reducing the deposition of this modification led to the downregulation of NC genes and the impairment of cell migration (Merkuri et al., 2024). These findings define an epigenetic mechanism that integrates lysine lactylation with the GRNs that orchestrate embryonic development.

### 4.4 Mammalian cell reprogramming

Histone lactylation has also emerged as a crucial epigenetic factor in cellular reprogramming, directly linking metabolic shifts to gene expression regulation. Its function in both *Glis1* and *Dux* during early reprogramming is exemplified. Glis1 (abbreviation of Gli-like transcription factor 1) is a newly identified reprogramming factor, also known to induce somatic cell reprogramming (Maekawa et al., 2011). Li and colleagues demonstrated that Glis1 directly binds to and opens chromatin at glycolytic genes during the early phases of reprogramming, whereas it closes chromatin at somatic genes. This dual action facilitates reprogramming by inducing a metabolic shift from oxidative phosphorylation to glycolysis, thereby increasing lactate production. This elevation in intracellular lactate enhances histone lactylation, subsequently activating key pluripotent genes such as Oct4 and Sall4 (Li L. et al., 2020). This cascade ultimately facilitates efficient cell reprogramming.

Similarly, *Double homeobox protein* (*Dux*) enhances reprogramming efficiency through promoting histone lactylation during the initial stages of induced pluripotent stem cell (iPSC) formation (Hendrickson et al., 2017). In mouse embryonic fibroblasts (MEFs), Hu et al. revealed that *Dux* recruits p300 via the C-terminal region, which significantly upregulated the level of H3K18 lactylation. Elevated levels of H3K18 lactylation regulate the transition from oxidative phosphorylation to glycolysis, thereby creating a metabolic environment conductive to reprogramming efficiency (Hu et al., 2024) (Figure 4B). These studies emphasize the key role of histone lysine lactylation as an initiator of reprogramming in early cell reprogramming.

### 4.5 Cardiovascular diseases (myocardial infarction)

Myocardial infarction (MI) triggers a complex immune response that is crucial for acute injury and post-infarction repair. It is characterized by the recruitment and activation of monocytes and macrophages (Fan M. et al., 2023). Wang and colleagues highlighted the role of histone lactylation in post-MI cardiac repair (Wang et al., 2022). Their study demonstrated a significant early increase in H3K18 lactylation within monocytes and macrophages, enhancing the transcription of reparative genes such as *Lrg1*, *Vegf-a*, and *IL-10*. These genes promoted anti-inflammatory and proangiogenic activities, creating an environment conducive to tissue regeneration and improved cardiac function after MI (Figure 4C) (Wang et al., 2022). This finding suggest that histone lactylation as a potential epigenetic regulator with therapeutic implications for cardiac repair.

Conversely, lactate can increase cardiac fibrosis and exacerbate cardiac dysfunction, through pathways such as Endothelial-to-Mesenchymal Transition (EndoMT) following MI (Fan M. et al., 2023). Fan et al. revealed that lactate induces EndoMT via lactylation of Snail, mediated by CBP/p300 enzyme and monocarboxylate transporter (MCT) -dependent signaling. Inhibiting Snail1 lactylation mitigates lactate-induced EndoMT and TGF-β/Smad2 activation after hypoxia/MI. These findings highlight lactate’s dual role in cardiac repair and dysfunction, emphasizing the need to balance its reparative and pathological impacts for better therapeutic outcomes.

### 4.6 Tumorigenesis

The Warburg effect, one of the hallmarks of tumors, produces large amounts of lactate due to the metabolism of glucose via glycolysis. Lactate from tumors contributes to tumor growth and progression by promoting protein lactylation, which regulation of gene expression via an epigenetic modification in cancer cells (Vander Heiden et al., 2009; Palsson-McDermott and O'Neill, 2013).

Zong et al. analyzed the TCGA breast cancer dataset and found that serum lactate levels were elevated in patients with wild-type p53, suggesting a direct role of tumor-derived lactate in the regulating p53 function (Zong et al., 2024). Further studies using a breast cancer mouse model discovered that tumor-derived lactate can promote p53 lactylation and is a natural inhibitor of p53. This process is conducted by AARS1, which acts as a lactate sensor, binding lactate and catalyzing the formation of lactate-AMP. This lactate-AMP is then transferred to lysine residues in the DNA-binding domain of p53, impairing its ability to bind DNA and attenuate its tumor-suppressive activity (Figure 4D). Interestingly, β-alanine competes with lactate for binding to AARS1, thereby preventing p53 lactylation and offering potential therapeutic opportunities for improving chemotherapy (Zong et al., 2024). However, considering the dual functionality of AARS1 in protein translation and lactylation, targeted inhibition strategies should be employed judiciously.

Lactate is secreted into the tumor microenvironment (TME), where it contributes to immune suppression by altering T-cell function (Certo et al., 2021). In tumor-specific cytolytic T lymphocytes (CTLs), lactate drives histone lactylation, which activates the transcription of *circATXN7*. This circular RNA interacts with NF-κB p65 subunit, sequestering it in the cytoplasm and impeding its nuclear signaling, making tumor-specific CTLs more susceptible to activation-induced cell death (AICD) and thereby reducing their ability to eliminate tumor cells (Figure 4E). Zhou et al. demonstrated that the upregulation of circATXN7 is associated with poor clinical outcomes and resistance to immunotherapy (Zhou et al., 2024). Targeting circATXN7 in T cells may offer a novel strategy to prevent tumor-mediated immune suppression and improve responses to immunotherapy.

Lactate also alters the immune landscape by affecting tumor-associated macrophages (TAMs). Li X.-M. et al. (2024) found that tumor-derived lactate promotes H3K18 lactylation in TAMs, inhibiting the expression of RARγ, a nuclear receptor that suppresses NF-κB signaling. This mechanism leads to the persistent activation of NF-κB, increasing interleukin-6 (IL-6) levels in the TME and enhancing the tumor-promoting functions of macrophages via STAT3 signaling (Figure 4F) (Li X.-M. et al., 2024). These findings reveal a new mechanism by which lactate-driven macrophage functional remodeling supports tumorigenesis. Furthermore, the authors identified nordihydroguaiaretic acid (NDGA) as a promising compound that directly targets RARγ, disputing inflammation-associated tomor growth in the TME. This discovery underscores the potential of targeting lactate-driven pathways as therapeutic strategies for cancer, specifically by modulating macrophage signaling and immune suppression.

## 5 Discussion and prospects

Lysine lactylation is a novel protein PTM and derived from lactate and other lactyl molecules. It plays a significant role in regulating various physiological and pathological processes, such as nervous system diseases (Pan et al., 2022) and tumor development (Chen et al., 2024). Although there have been significant advances on lactylation regulation and its functions, the study of lysine lactylation is still at an early stage and several issues need to be further addressed.

Lysine lactylation exists in three isomeric forms: K_L-la_, K_D-la_, and K_ce_. Among these, K_L-la_ is the most extensively studied, primarily because it is the most prevalent lactylation type on histones in eukaryotes (Zhang et al., 2024). This predominance may stem from the high abundance of L-lactate in eukaryotic systems and the development of specific detection techniques and anti-K_L-la_ antibodies. Conversely, prokaryotes predominantly generate D-lactate as their primary form of lactate (Remund et al., 2023). Previous studies have shown that D-lactate, derived from gut microbes, is transported via the portal vein into the liver, where it triggers Kupffer cells to recognize and kill pathogens (McDonald et al., 2020). Recently, evidence has indicated that D-lactate also plays a role in modulating M2 tumor-associated macrophages and remodeling of the immunosuppressive tumor microenvironment in hepatocellular carcinoma (Han et al., 2023). This suggests that K_D-la_ as the primary lactylation form in prokaryotes and its potential function in intestinal diseases and cancer. However, lysine lactylation currently found in prokaryotes are still in the form of K_L-la_ (Dong et al., 2022; Li et al., 2023). Further research is needed to identify K_D-la_ in prokaryotes and its potential roles in host-microbe interactions.

Currently, three primary techniques are used to identify lysine lactylation: antibody-based immunoprecipitation, metabolic labeling, and computational prediction. Each method has its unique strengths and limitations. Antibody-based immunoprecipitation relies on specific antibodies against endogenous PTM. This approach has recently seen advances with the development of anti- K_D-la_ antibodies, which are critical for studying lysine lactylation in prokaryotes. Moreover, antibody specificity remains a significant challenge—non-specific binding can lead to high background contamination, compromising data reliability (Li et al., 2022). Metabolic labeling incorporates bio-orthogonal chemical analogs of PTM donor precursors into modified proteins, using the endogenous translation machinery of model organisms. This method enables global analysis but comes with challenges: the analogs must closely resemble the structure of PTM donor precursors, posing potential experimental complexity and toxicity risks (Saleh et al., 2019). Computational prediction tools offer a cost-effective alternative without requiring expensive reagents or complex protocols. While these tools are invaluable for initial identification, they require experimental validation to confirm predictions, as their accuracy remains dependent on high-quality datasets and robust algorithms (Jiang et al., 2021; Lv et al., 2022; Lai and Gao, 2023; Yang et al., 2024). Future research might address these limitations by combining the strengths of different approaches. For example, integrating antibody-based techniques with computational predictions may improve identification specificity. Additionally, advancements in techniques for isolating bacterial cells under diverse environmental conditions could provide insights into the prokaryotic lactylome and its conservation in various biological systems (Ren et al., 2017).

The regulation of lysine lactylation involves several key enzyme families. In eukaryotes, p300/CBP and MYST families are the primary enzymes responsible for adding lactyl groups to lysine residues. Conversely, the GNAT and AARS enzyme families regulate lysine lactylation across in prokaryotes and eukaryotes. On the other hand, enzymes such as HDAC1/3 and SIRT1-3 remove lactyl groups from lysine residues (Fu et al., 2023). However, the exact roles of these enzymes across different organisms remain unclear. Therefore, in-depth research is needed to clarify their specific contributions to lysine lactylation.

Moreover, the relationship between lactylation and other PTMs has been explored. For instance, lactylation represents a distinct PTM compared to acetylation (Li P. et al., 2020) and crotonylation, with unique effects on cellular processes such as gene regulation and homeostasis (Cao et al., 2024; Yao et al., 2024). Studies have shown that lactate can drive both lactylation and acetylation of HMGB1 through p300/CBP in macrophages. Yang et al. also reported that lactate enhances HMGB1 acetylation via GPR81 and Hippo/YAP mediated pathways. Reducing lactate production or inhibiting GPR81 signaling *in vivo* was shown to decrease exosomal HMGB1 and improve survival in polymicrobia sepsis (Yang et al., 2022). Sun et al. found that lactylation competitively inhibits PARP1 acetylation, resulting in recovery of its ADP-ribosylation activity and promoting DNA repair (Sun et al., 2022a). Additionally, lactylation has demonstrated crosstalk with other PTMs. For instance, Dai et al. reported differences in the distribution of histone crotonylation and lactylation within brain tissue, suggesting these PTMs interact to regulate gene expression during different stages of neural development (Dai et al., 2022). These findings do not only illustrate the complexity of PTM interplay but also highlight gaps in our understanding of the mechanisms of their interactions.

Nevertheless, the study of lysine lactylation is expected to achieve breakthroughs in the near future in key areas. In basic research, in-depth exploration of the regulatory mechanism of lysine lactylation, especially its interaction with other PTMs, will help researchers better understand how lactylation participates in the physiological and pathological processes of life. In applied research, increased focus on specific activators and inhibitors of lactylation are expected to provide strong support for the development of new drugs.
